# Human Exposure to Toxic Metals (Al, Cd, Cr, Ni, Pb, Sr) from the Consumption of Cereals in Canary Islands

**DOI:** 10.3390/foods10061158

**Published:** 2021-05-21

**Authors:** Carmen Rubio-Armendáriz, Soraya Paz, Ángel J. Gutiérrez, Dailos González-Weller, Consuelo Revert, Arturo Hardisson

**Affiliations:** 1 Department of Toxicology, Universidad de La Laguna, La Laguna, 38200 Tenerife, Canary Islands, Spain; crubio@ull.edu.es (C.R.-A.); spazmont@ull.edu.es (S.P.); dgonzal@ull.edu.es (D.G.-W.); atorre@ull.edu.es (A.H.); 2 Health Inspection and Laboratory Service, Canary Health Service, S/C de Tenerife, 38006 Tenerife, Canary Islands, Spain; 3 Departament of Physical Medicine and Pharmacology, University of La Laguna, 38200 Tenerife, Canary Islands, Spain; mgirones@ull.edu.es

**Keywords:** Canary Islands, cereals, corn, roasted corn, corn gofio, gofio, barley gofio, flour, wheat, toxic metals, risk assessment

## Abstract

The Canary Islands is an archipelago that consumes cereals and derivatives imported from other regions of the world. The increase in contamination with toxic metals makes it necessary to assess the content of toxicological metals of interest to ensure quality and safety. The content of toxic metals (Al, Cd, Cr, Pb, Ni, Sr) was determined in 221 samples of cereals and derivatives (corn, wheat, gofio, corn gofio, barley gofio, roasted corn and flour) marketed in the Canary Islands using ICP-OES (inductively coupled plasma optical emission spectrometry) to assess dietary exposure. Al content recorded in barley gofio (29.5 mg/kg fresh weight) stands out. The estimated daily intake (EDI) of Pb is 52 µg/day if 100 g/day of barley gofio is consumed (121% and 240% of the BMDL nephrotoxicity limit set by the EFSA at 0.63 µg/kg body weight/day for adults and children, respectively). The EDI of PB is 16 µg/day if 30 g barley gofio/day is consumed by adults (36.2% of the abovementioned BMDL nephrotoxicity limit). The EDI of Pb is 7.8 µg/day if 15 g barley gofio/day is consumed by children (32.2% of the abovementioned BMDL nephrotoxicity limit). Gofio is a food of high nutritional value. It is necessary to establish monitoring programs for toxic metals in raw materials and processed products to reduce exposure levels.

## 1. Introduction

**Figure 1:**
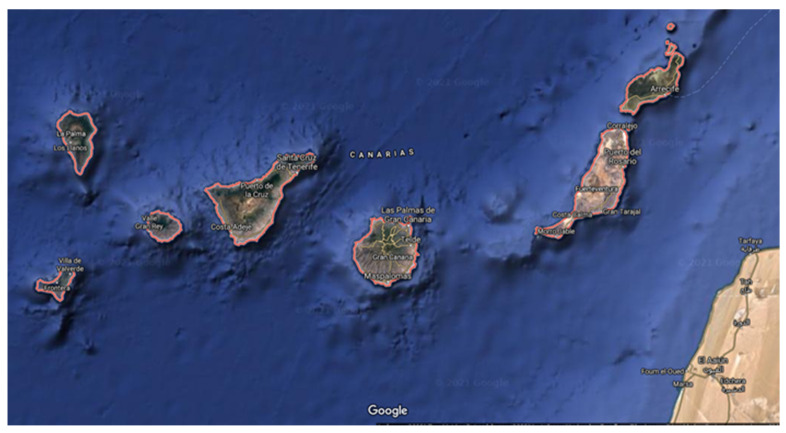
Canary Islands (Source: Google Maps™).

The archipelago of the Canary Islands is made up of western islands El Hierro, La Gomera, La Palma and Tenerife and of eastern islands Gran Canaria, Fuerteventura, Lanzarote and La Graciosa (Figure 1). The Canary archipelago is located in northwest Africa near the coast of Morocco and the north of Western Sahara. It belongs to the Macaronesia region along with Cape Verde, Azores, Madeira and the Savage Islands. According to data from the Official State Gazette published in December 2020, the population of the Canary Islands amounts to 2,175,952 inhabitants [1].

Cereals have been the basis of human nutrition since ancient times [2]. In the Canary Islands, the consumption of cereals, especially in the form of gofio, was the basic sustenance from the aborigines who populated the islands until long after the Conquest by the Spaniards.

Canarian gofio is a food that has a protected geographical indication (PGI) and is obtained in the Canary archipelago, resulting from the grinding of toasted cereals with or without the addition of sea salt. Sometimes gofio can be complemented with legumes that are subjected to the same treatment as cereals. The product obtained has an appearance similar to wholemeal flours since it presents in the form of a floury powder [3,4,5].

Although cereals, and especially gofio, play an important role in the diet of the residents of the Canary Islands, urbanization of the Canary Islands has caused changes in the consumption habits of the islanders. Thus, as a consequence of increasing urbanization, the consumption of processed foods has increased considerably [6]. It is estimated that in 2013, the consumption of cereals in the Canary Islands was 173.7 g per person per day [6].

Cereals, especially whole grains and their processed derivatives, are foods of high nutritional value due to their mineral content, dietary fiber and complex carbohydrates [7,8,9,10]. However, due to high anthropogenic contamination of crops, numerous studies have been published that report data on toxic metals in cereals for human consumption [11,12].

Among the different toxic or potentially toxic metals that can be found in cereals, aluminum (Al), cadmium (Cd), chromium (Cr), nickel (Ni), lead (Pb) and strontium (Sr) stand out.

Toxic metals such as Al, Cd and Pb are environmental pollutants originating mainly from anthropogenic activities such as mining, uncontrolled use of pesticides or from spills from industries in which they are used. These metals are toxic even in small amounts. Their main problem is high tendency to accumulate [13]. Cereals are among the main dietary sources of these toxic elements.

Al tends to accumulate in the brain and is associated with neurodegenerative diseases, although its absorption through the gastrointestinal tract is low (0.1%) [14]. Cd is an element that competes with essential divalent elements such as Zn, Cu or Fe and, in addition, it is a nephrotoxic agent that tends to accumulate in the kidneys; in high concentrations, it can cause renal failure [15]. Pb is a neurotoxic element that can cause damage to the central nervous system (CNS), especially in developing children and fetuses [16].

Other metals of toxicological interest such as Cr, Ni or Sr are associated with adverse effects in case of high intake. It should be noted that, in the case of these elements, their presence in food, especially in cereals, is not only due to anthropogenic factors, but rather that plant organisms need elements such as Cr or Ni that are involved in their metabolism and life cycle.

Cr, in case of high and prolonged intake, can trigger chronic renal failure, dermatitis, bronchitis, asthma, etc. [17,18,19].

The relationship between Ni and the enzyme urease makes it an essential element for plants [20,21]. Notable changes in body weight have been observed in experimental animals. Individuals with hypersensitivity to Ni or with kidney problems are susceptible to damage by ingestion of Ni [17,22].

High intake of Sr is related to phosphorus deficiency since these elements compete with each other; its accumulation in bones could lead to an increase in bone density [23].

Nawab et al. [24] studied the content of toxic metals in cereals from Pakistan, registering levels that exceeded the recommendations established by the FAO/WHO. More recently, Khaneghah et al. [25] studied the content of toxic metals in cereals and cereal-based products, highlighting the concentrations of Cu, Cd, Pb, Fe, Hg and Mn in flour. These publications demonstrate the importance of controlling the levels of toxic metals in cereals and derivatives to protect health of consumers.

The objectives of this study are (i) to determine the content of toxic metals (Al, Cd, Cr, Ni, Pb, Sr) in samples of cereals and derived products (corn, wheat, gofio, corn gofio, barley gofio, gofio wheat, roasted corn and flour) marketed in the Canary Islands and (ii) to assess the dietary exposure of the Canarian population based on the consumption of these cereals.

## 2. Material and Methods

### 2.1. Samples and Sample Treatment

A total of 221 samples of cereals (corn, wheat) and derivatives (gofio, corn gofio, barley gofio, roasted corn and flour) (Table 1) marketed in the Canary Islands (Figure 1) were analyzed. The samples of cereals and their derivatives were collected from the islands of Tenerife, Gran Canaria, La Palma and Fuerteventura.

The samples were collected monthly (2017–2019) from supermarkets of the Canary Islands. Currently, the Canary Islands have a total of 40 gofio mills. Gofio samples were collected from 20 mills located in the metropolitan areas (*n* = 11) of Tenerife and Gran Canaria and in different towns (*n* = 9) of La Palma and Fuerteventura.

Collection of the samples was carried out in accordance with the provisions of Royal Decree 538/2015 of June 26 which regulates the performance of studies, reports and comparative analyses regarding food products [26]. The samples were collected in triplicate, ensuring the same batch whenever possible, noting the identification data of each sample (type, place and date of collection, quantity, batch, storage conditions, operator who collected the sample). The samples were collected in sterile plastic containers (sample collection bags) and sealed until they were opened in the laboratory. The collected samples were commercial samples (featured the same conditions as the ones sold to the general public).

It should be noted that, as it is a research project and not an official analysis, the samples were not transferred to food safety agencies; instead, the three replicates were analyzed in the laboratory.

An analytical precision balance (Balance XPR204S, Mettler Toledo, Columbus, OH, USA) was used to weigh 1 g of the homogenized sample, in triplicate, into pressure vessels (HVT50, Anton Paar, Graz, Austria). To proceed with the digestion process, we added 4 mL of 65% HNO_3_ (Sigma-Aldrich, Steinheim, Germany) and 2 mL of H_2_O_2_ (Sigma-Aldrich, Steinheim, Germany) to the pressure vessels containing the samples, and then the pressure vessels were closed and placed in a microwave oven (Multiwave Go Plus, Anton Paar, Graz, Austria). The digestion conditions are described in the Table 2.

The samples digested were poured into a 10-mL volumetric flask with Milli-Q distilled water, and then the samples were transferred to PET containers until determination.

### 2.2. Analytical Method

Inductively coupled plasma optical emission spectrometry (ICP-OES) (ICAP 6300 Duo, Thermo Scientific, Waltham, MA, USA) was used to determine the content of toxic metals (Al, Cd, Cr, Ni, Pb, Sr) with the following instrumental conditions: 1150 W (RF power); 0.5 L/min (gas flow); 50 rpm (injection of the sample to the flow pump); 0 s (stabilization time).

The instrumental wavelengths of the analyzed elements were as follows: Al (167.0 nm), Cd (226.5 nm), Cr (267.7 nm), Pb (220.3 nm), Ni (231.6 nm) and Sr (407.7 nm). The LOQ (limits of quantification) obtained as per the IUPAC instructions [27] were as follows: Al (0.012 mg/L), Cd (0.001 mg/L), Cr (0.008 mg/L), Pb (0.001 mg/L), Ni (0.003 mg/L) and Sr (0.003 mg/L). Table 3 shows the recovery percentage (RP) obtained under reproducible conditions with a certified reference material (RP > 94%).

### 2.3. Statistical Analysis

Statistical analysis was conducted using IBM Statistics SPSS 24.0 (Armonk, NY, USA) for Windows ™. The value of *p* < 0.05 was considered to indicate a significant difference. The statistical study was carried out considering the content of toxic metals (Al, Cd, Cr, Pb, Ni, Sr) in the different types of cereals and products analyzed (corn, wheat, gofio, corn gofio, barley gofio, roasted corn and flour).

The normality of concentration of toxic metals was studied using the Kolmogorov–Smirnov and Shapiro–Wilk tests. The Levene test was applied to the homogeneity of variance study. The data followed a non-normal distribution, and the Kruskal–Wallis non-parametric test was applied [12].

### 2.4. Dietary Intake Calculation

The estimated daily intake (EDI) is defined as the amount of a chemical element/substance/contaminant that is ingested with a portion of a food or foods (Equation (1)). The calculation of this value makes it possible to evaluate the risk when comparing it with the reference value established by food safety institutions. The contribution percentage (Equation (2)) allows obtaining a percentage value over the reference value.

(1) $$\mathrm{EDI}\;\left(\frac{\mathrm{mg}}{\mathrm{day}}\right)=\;\mathrm{Cereal}\;\mathrm{consumption}\;\left(\frac{\mathrm{kg}}{\mathrm{day}}\right)\;\times \;\mathrm{Toxic}\;\mathrm{metal}\;\mathrm{concentration}\;\left(\mathrm{mg}/\mathrm{kg}\;\mathrm{fresh}\;\mathrm{weight}\right)$$

(2) $$\mathrm{Contribution}\;\mathrm{percentage}\;\left(\%\right)=\left[\frac{\mathrm{EDI}}{\mathrm{Reference}\;\mathrm{value}}\right]\;\times \;100\;$$

Table 4 lists the reference values that were used for the exposure assessment. These values were set by reference institutions such as the European Food Safety Authority (EFSA) and the World Health Organization (WHO). Likewise, an adult weight of 68.48 kg and a child weight of 38.48 kg were considered as established by the AECOSAN (Spanish Agency for Consumption, Food Safety and Nutrition) [28].

## 3. Results and Discussion

### 3.1. Content of Toxic Metals in Cereals and Derivative Products

Table 5 shows the mean concentrations of toxic metals in the different cereals and derivatives analyzed.

In general terms, the toxic metals analyzed follow the descending order of Al > Sr > Ni > Pb > Cr > Cd. Al stands out as the metal with the highest concentration in all the analyzed cereals and derivatives, registering the highest average concentrations in barley gofio (29.5 mg/kg fresh weight) and in corn (28.1 mg/kg fw). The studies carried out by Brizio et al. [32] recorded an average content of Al in barley of 11.43 mg/kg, with a maximum concentration of 1009 mg/kg, which shows that this cereal tends to accumulate large concentrations of Al. Likewise, various studies have shown a clear relationship between the low pH of the soil and higher concentrations of Al in them, which later pass to the crops that are cultivated in these soils [33,34].

Sr is another toxic metal that stands out; again, it is barley gofio that registered the highest average concentration (1.46 mg/kg fw), followed by flour with an average concentration of 0.955 mg/kg fw. The studies carried out by Rubio-Armendáriz et al. [12] report a similar concentration of Sr in wheat samples (1.60 mg/kg). On the other hand, the highest Ni content was detected in mixed gofio (0.351 mg/kg fw).

Again, barley gofio is the derivative that registered the highest concentrations of Pb (0.520 mg/kg fw) and Cr (0.280 mg/kg fw). Millour et al. [35] recorded a mean Pb concentration of 0.006 mg/kg in breakfast cereals, which is lower than that recorded in the present study. However, it should be noted that gofio is a derivative that is made from whole toasted cereal including the peel, which is where the highest concentration of toxic metals accumulates [3,12,36]. Considering the current legislation [37] that regulates the content of toxic metals in foods for human consumption, it was found that barley gofio (0.520 mg/kg fw) exceeds the Pb limit set for cereals (0.20 mg/kg), which is why it would not be suitable for consumption.

Wheat registered the highest average concentration of Cd (0.04 mg/kg fw). Tejera et al. [38] registered a similar concentration of Cd in wheat flour of 0.027 mg/kg, which is slightly lower than the level registered in wheat in this study.

The statistical study showed the existence of significant differences (*p* < 0.05) in the levels of Al in corn gofio, of Sr in corn and of Ni in flour with the rest of the samples analyzed.

### 3.2. Adult Dietary Intake Assessment

Table 6 shows the estimated daily intake (EDI) values and the percentage of contribution to the reference values (see “Dietary Intake Calculation”) for adults.

Barley gofio, gofio and corn gofio stand out for their dietary contributions of Pb. The EDI of Pb is 52 µg/day if 100 g/day of barley gofio is consumed. The BMDL (benchmark dose level) value established by the EFSA at 0.63 µg/kg body weight/day (nephrotoxic effects) is widely exceeded with the intake of 100 g/day of barley gofio (121% of BMDL_nephrotoxicity_). Generally speaking, gofio consumption should be limited to about 30 g/day (one serving) [3], which would represent a lower contribution percentage (36.3% of BMDL_nephrotoxicity_). However, Pb is described as one of the most common contaminants in cereals, these products being the main source of exposure to Pb [39]. It is necessary to study the possible sources of contamination that increase the level of Pb in the gofio. It should be noted that this Pb contamination is not due in itself to the raw materials, but to the company that produces the gofio. The studies carried out by Caballero-Mesa [40] show a clear relationship between the production industry and the final contamination of the gofio with Pb.

Gofio is a food with high nutritional value. However, the persistence of some gofio mills in the Canary Islands where some parts of the production process are neglected raises the level of Pb in the final product. It is necessary to establish monitoring programs for the levels of Pb in the raw materials and in the processed products in order to reduce the levels of dietary exposure to Pb. 

It should be noted that, considering the EFSA report on dietary Pb exposure in the European population, the mean Pb intake for an adult is 0.50 µg Pb/kg bw/day [41]. This means that, considering the average weight of an adult established by the AECOSAN, the average intake of Pb would be 34.2 µg Pb/day. However, the intake of Pb from the consumption of 100 g/day of the gofio analyzed would be 52 µg Pb/day, exceeding the European average. However, if we consider normal consumption of a portion of gofio (30 g/day) [3], the intake of Pb would be 16 µg Pb/day from barley gofio consumption, which is, in this case, well below the European average.

Likewise, the EDI of Al is 2.95 mg/day if 100 g/day of barley gofio is consumed (30.2% of the TWI of Al set at 1 mg/kg bw/week by the EFSA) [14]. This percentage is high, especially considering that Al is an element that is found in all foods in variable concentrations. That is, barley gofio provides a large contribution of Al to the global intake of this element. Similarly, corn stands out because its consumption (100 g/day) contributes 28.7% of the TWI of Al mentioned above. In case of a normal portion of the gofio (30 g/day) [3], the intake of Al would be 885 µg Al/day from barley gofio consumption with the contribution percentage of 9.1% of the abovementioned TWI value. Therefore, it is necessary to regulate the content of this metal in cereals by setting maximum concentration values.

### 3.3. Children Dietary Intake Assessment

Table 6 shows the EDI values and the percentage of contribution to the reference values (see “Dietary Intake Calculation) for children.

The gofio analyzed, including barley, wheat and mixed gofio, contributes significantly to the intake of Pb, Al and Cd. The consumption of 100 g/day of barley gofio represents a contribution percentage of 240% to the fixed Pb BDML for nephrotoxic effects (0.63 µg/kg bw/day). Likewise, 100 g/day of gofio (70.5% of the BMDL of Pb) and corn gofio (40.6% of the BMDL of Pb) may be considered large contributions to the BMDL of Pb for nephrotoxic effects.

As mentioned above, these data are worrying because, considering a prolonged consumption of 100 g/day of any of the gofio samples analyzed, children would be at risk due to high Pb intake. However, in case of moderate consumption of a standard ration of gofio (15 g/day) [3], the maximum intake value set for Pb is not exceeded, with contribution percentages of 32.2% (barley gofio), 9.5% (gofio) and 5.6% (corn gofio) to the BDML of Pb for nephrotoxic effects (0.63 µg/kg bw/day). It is, therefore, necessary to moderate the rations of barley gofio since it seems to be the one that offers the greatest contribution to the BMDL of Pb to avoid dietary overexposure to Pb in the case of children between 7 and 12 years of age.

The EFSA report on dietary Pb exposure in the European population reports an exposure of 1.03 µg Pb/kg bw/day in children [41]. Considering the average weight set by the AECOSAN for children between 7 and 12 years old, the intake of Pb would be 35.5 µg Pb/day. However, the daily Pb intake from the consumption of 100 g/day of barley gofio analyzed would be 52 µg Pb/day, far exceeding the average Pb intake established for European children. However, considering the consumption derived from a gofio ration (15 g/day) [3], the intake would be 7.8 µg Pb/day in the case of barley gofio, which is lower than the European average.

EDI, estimated daily intake when consuming 100 g/day. Mean average weight of adults—68.48 kg, children—34.48 kg [28]. Based on the BMDL to nephrotoxicity^1^ and cardiovascular effects^2^, 100 g/day of barley gofio and corn gofio confer Al contribution percentages of 59.9% and 57.0%, respectively, to the TWI of Al (1 mg/kg bw/week). Although these values do not exceed 100% of the TWI, it is necessary to consider this contribution significant. However, in case of consumption of a normal portion of gofio (15 g/day) [3], contribution percentages of 8.1% (barley gofio) and 1.9% (corn gofio) of the TWI of Al (1 mg/kg bw/week) would be obtained, so child consumers (7–12 years of age and 34.48 kg bw) would not be at risk.

The percentage of contribution of the consumption of 100 g/day of wheat (32.5% of the TDI of Cd established at 2.5 µg/kg bw/week) is also noteworthy. However, considering that cereals are among the foods that most contribute to the dietary intake of Cd [42], this result is within expectations.

## 4. Conclusions

Barley gofio stands out for registering the highest concentrations of Al, Sr, Pb and Cr. It should be noted that the Pb content registered in barley gofio exceeds the legal limit established by Commission Regulation (EC) No 1881/2006 of 19 December 2006 setting maximum levels for certain contaminants in foodstuffs, not being suitable for consumption. This fact highlights the need to establish monitoring and surveillance programs for cereals and their derivatives in the Canary Islands to detect possible non-compliance and, therefore, avoid their use.

The need to assess the dietary exposure of the Canarian population not only to the consumption of cereals and derivatives, but also to other foods that can be considered a source of toxic metals should also be noted. Therefore, it is necessary to continue with research work in this area to preserve the health of the population of Canary Islands.

## Figures and Tables

**Table 1:** Characteristics of the analyzed samples.

Sample	No. of Samples	Origin
Gofio	37	Spain, Bulgaria and Argentina
Barley gofio	5	Spain
Corn gofio	29	Spain, Bulgaria and Argentina
Flour	9	Spain
Corn	81	Spain, Bulgaria, France and Argentina
Toasted corn	56	Spain, Bulgaria and Argentina
Wheat	4	Spain
Total	221	

**Table 2:** Instrumental conditions of the microwave digestion process.

No.	Ramp (min)	Temperature (°C)	Time (min)
1	15	50	5
2	5	60	4
3	5	70	3
4	3	90	2
5	20	180	10

Microwave processing power, 850 W; maximum temperature, 200 °C; cooling temperature, 50 °C.

**Table 3:** Recovery study results based on the recovery percentage (RP) with reference materials.

Metal	Certified Material	Concentration Recorded (mg/kg)	Concentration Certified (mg/kg)	RP (%)
Al	SRM 1515 Apple Leaves	286 ± 9	285.1 ± 26	99.7
Sr		25.0 ± 2.0	24.6 ± 4.0	98.3
Cr		0.29 ± 0.03	0.30 ± 0.00	97.8
Ni	SRM 1548a Typical Diet	0.37 ± 0.02	0.38 ± 0.04	102.3
Pb		0.044 ± 0.000	0.044 ± 0.013	98.9
Cd	SRM 1567a Wheat Flour	0.026 ± 0.002	0.026 ± 0.008	98.4

**Table 4:** Intake limit values of the toxic metals analyzed (Al, Cd, Cr, Pb, Ni, Sr).

Toxic Metal	Parameter	Intake Limit Values	Organization	References
Cr(III)	TDI	0.3 mg/kg bw/day	EFSA	[19]
Ni		13 µg/kg bw/day		[29]
Pb	BMDL	0.63 µg/kg bw/day ^1^ 1.50 µg/kg bw/day ^2^		[30]
Sr	TDI	0.13 mg/kg bw/day	WHO	[31]
Al	TWI	1 mg/kg bw/week	EFSA	[14]
Cd		2.5 µg/kg bw/week		[15]

EFSA, European Food Safety Authority; WHO, World Health Organization; BMDL, benchmark dose level; TDI, tolerable daily intake; TWI, tolerable weekly intake; bw, body weight; nephrotoxicity ^1^ and cardiovascular effects ^2^.

**Table 5:** Mean concentrations and standard deviations (mg/kg fresh weight ± SD) of the analyzed toxic metals by cereal and cereal derivative type.

Type	Al	Cd	Cr	Ni	Pb	Sr
Gofio	4.89 * ± 5.49	0.005 ± 0.01	0.054 ± 0.07	0.351 ± 0.46	0.153 ± 0.20	0.827 ± 1.27
Barley gofio	29.5 ± 0.00	0.010 ± 0.00	0.280 ± 0.00	0.150 ± 0.00	0.520 ± 0.00	1.46 ± 0.00
Corn gofio	6.90 ± 3.67	0.003 ± 0.01	0.102 ± 0.05	0.209 ± 0.07	0.090 ± 0.12	0.345 ± 0.17
Flour	2.93 ± 1.13	0.019 ± 0.01	0.010 ± 0.03	0.05* ± 0.08	0.054 ± 0.05	0.273 ± 0.11
Corn	28.1 ± 231	0.001 ± 0.006	0.081 ± 0.08	0.264 ± 0.40	0.050 ± 0.05	0.208 * ± 0.15
Toasted corn	2.92 ± 2.40	0.002 ± 0.006	0.062 ± 0.07	0.289 ± 0.36	0.051 ± 0.06	0.427 ± 0.67
Wheat	8.18 ± 3.71	0.040 ± 0.00	0.165 ± 0.06	0.140 ± 0.03	0.070 ± 0.00	0.955 ± 0.53

* Statistical difference (*p* < 0.05).

**Table 6:** Assessment of dietary intake of toxic metals based on the EDI (mg/day) and the contribution percentage (%) for adults and children.

**Adults**	**EDI**	**%**	**EDI**	**%**	**EDI**	**%**	**EDI**	**%**	**EDI**	**%**	**EDI**	**%**	**EDI**	**%**
	**Gofio**		**Barley Gofio**		**Corn Gofio**		**Flour**		**Corn**		**Toasted Corn**		**Wheat**	
Al	0.489	5.00	2.95	30.2	0.690	7.05	0.293	3.00	2.81	28.7	0.292	2.99	0.818	8.36
Cd	0.0005	2.10	0.001	4.09	0.0003	1.13	0.002	7.72	0.0001	0.50	0.0001	0.73	0.004	16.4
Cr	0.05	0.03	0.028	0.14	0.010	0.05	0.001	0.00	0.026	0.04	0.006	0.03	0.017	0.08
Ni	0.035	3.94	0.015	1.68	0.021	2.34	0.005	0.57	0.026	2.96	0.029	3.25	0.014	1.57
Sr	0.083	0.93	0.146	1.64	0.034	0.39	0.027	0.31	0.021	0.23	0.043	0.48	0.096	1.07
Pb ^1^	0.015	35.5	0.052	121	0.009	20.5	0.005	12.6	0.005	11.5	0.005	11.9	0.007	16.2
Pb ^2^	0.015	14.9	0.052	50.6	0.009	8.60	0.005	5.30	0.005	4.80	0.005	5.00	0.007	6.80
**Children**	**EDI**	**%**	**EDI**	**%**	**EDI**	**%**	**EDI**	**%**	**EDI**	**%**	**EDI**	**%**	**EDI**	**%**
	**Gofio**		**Barley Gofio**		**Corn Gofio**		**Flour**		**Corn**		**Toasted Corn**		**Wheat**	
Al	0.489	9.33	2.95	59.9	0.690	14.0	0.293	5.96	2.81	57.0	0.292	5.94	0.818	16.6
Cd	0.0005	4.17	0.001	8.12	0.0003	2.24	0.002	15.3	0.0001	1.00	0.0001	1.45	0.004	32.5
Cr	0.05	0.05	0.028	0.27	0.010	0.10	0.001	0.01	0.026	0.08	0.006	0.06	0.017	0.16
Ni	0.035	7.83	0.015	3.35	0.021	4.65	0.005	1.14	0.026	5.89	0.029	6.46	0.014	3.12
Sr	0.083	1.85	0.146	3.26	0.034	0.77	0.027	0.61	0.021	0.46	0.043	0.95	0.096	2.13
Pb ^1^	0.015	70.5	0.052	240	0.009	40.6	0.005	25.1	0.005	22.8	0.005	23.7	0.007	32.2
Pb ^2^	0.015	29.6	0.052	101	0.009	17.1	0.005	10.5	0.005	9.6	0.005	9.90	0.007	13.5

Nephrotoxicity ^1^ and cardiovascular effects ^2^.

## Data Availability

The datasets generated during the current study are not publicly available but are available from the corresponding author on reasonable request.
